# Crystallography: 1970 – 2025 and Beyond

**DOI:** 10.1063/4.0001074

**Published:** 2025-10-27

**Authors:** Carolyn P Brock

**Affiliations:** University of Kentucky, Lexington, KY, USA

## Abstract

As a graduate student I went to my first ACA meeting in 1970, and have been at an ACA or IUCr meeting in 52 of the last 55 years. During that time crystallography has developed in ways that could hardly have been imagined when I was a student.

In 1970 determining and reporting a single structure containing 30 independent non-H atoms was a task that often took months. Now the task usually takes only hours, thanks to the great advances in computer hardware and software. Furthermore, much larger structures, *e.g.* of proteins, are now routinely determined at atomic resolution.

The Cambridge Structural Database was founded in 1965 and the Protein Data Bank in 1971. As the number of entries became large and the software for using them sophisticated, the databases became research tools in their own right.

Structure determination at non-ambient temperatures has become routine. Crystals of very reactive materials can be stabilized by cooling. The passage of a crystal through a phase transition can be documented if the crystal remains single. Studies at non-ambient pressures are more difficult, but no longer unusual.

Decades ago diffraction patterns from powder samples were mostly used for identification. Now a structure can often be refined using the Rielveld method if the sample is pure, and if it is not, the phase ratios can often be determined.

Advances in X-ray sources have made it possible to measure diffraction patterns of very tiny crystals. Advances in neutron sources have made it possible to study magnetic structures and their phase transitions.

Structures of aperiodic crystals can now be determined and refined.

Enough has been learned that crystal design and crystal-structure prediction have become reasonable goals.

Recent advances in electron diffraction seem likely to open up whole new fields of study. We don’t yet know all the questions that AI will allow us to answer.

The future is bright even as it is being said in some circles that crystallography is a tool rather than a science. But that language is not new; it has been around for many decades. Challenging it seems to be a continuing necessity.

